# Silver nanoparticles reduce the apoptosis induced by tumor necrosis factor-α

**DOI:** 10.1080/14686996.2018.1487761

**Published:** 2018-07-16

**Authors:** Alaa Fehaid, Akiyoshi Taniguchi

**Affiliations:** a Cellular Functional Nanobiomaterials Group, Research Center for Functional Materials, National Institute for Materials Science, Tsukuba, Japan; b Graduate School of Advanced Science and Engineering, Waseda University, Tokyo, Japan; c Forensic Medicine and Toxicology Department, Faculty of Veterinary Medicine, Mansoura University, Dakahlia, Egypt

**Keywords:** Silver nanoparticles, tumor necrosis factor, apoptosis, cellular uptake, lung cell, 30 Bio-inspired and biomedical materials, 211 Scaffold / Tissue engineering / Drug delivery, 212 Surface and interfaces

## Abstract

Silver nanoparticles (AgNPs) are widely known to have anti-inflammatory properties, but the exact mechanism underlying this anti-inflammatory effect is not clearly understood. Tumor necrosis factor-α (TNFα) is a major pro-inflammatory cytokine that is expressed in the early stage of cell inflammation and induces apoptosis by several known pathways. Our study aimed to investigate the effect of AgNPs on the response of lung epithelial cells to TNFα and the molecular mechanism of this response. Lung epithelial cell line NCI-H292 cells were exposed to AgNPs (5 µg/mL) and/or TNFα (20 ng/mL) for 24 h, then cellular uptake was analyzed using flow cytometry. Our results showed that AgNPs were taken up by cells in a dose-dependent manner and that the cellular uptake ratio of AgNPs was significantly increased in the presence of TNFα. Apoptosis assays indicated that exposure to AgNPs significantly decreased the apoptotic effect of TNFα. Confocal microscopy was used to localize the tumor necrosis factor receptor 1 (TNFR1) and revealed that TNFR1 localized on the surface of cells exposed to TNFα. In contrast, TNFR1 localized inside cells exposed to both AgNPs and TNFα, with very few receptors scattered on the cell membrane. The results indicated that AgNPs reduced the cell surface TNFR1 expression level. The results suggested that the reduction of surface TNFR1 reduced cellular response to TNFα, resulting in an anti-apoptotic effect.

## GRAPHICAL ABSTRACT

**Figure:**
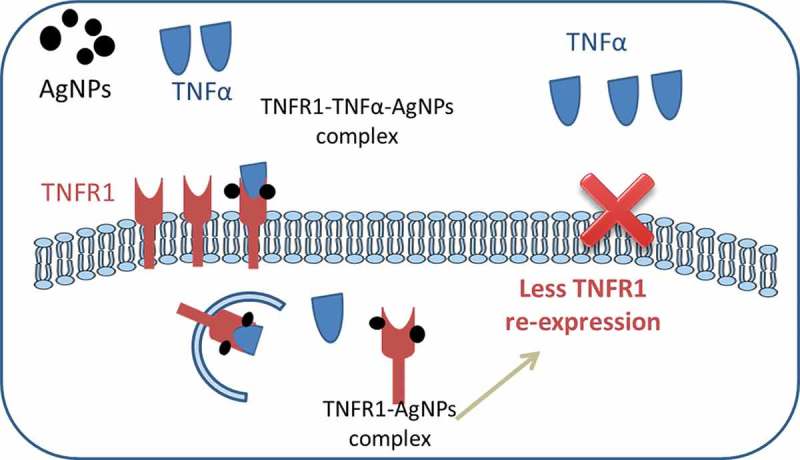

## 1. Introduction

The potent antimicrobial effect of silver nanoparticles (AgNPs) [1] has led to their incorporation into many products, including medical products such as wound dressings [2], catheters coatings, and surgical instruments [3]. AgNPs are also widely used in other consumer products such as deodorant sprays [4], fabrics [5], cosmetics, and electronics [6]. We are therefore likely exposed to AgNPs in our daily life and this has made AgNPs an important research topic. Our previous research demonstrated that the intratracheal instillation of 100 µg/rat of AgNPs caused subchronic inflammation in lung tissues, suggesting that a high concentration of AgNPs induces an inflammatory effect [7]. *In vitro* studies using a lung epithelial cell line as target cells is useful for elucidating more details regarding the molecular mechanism underlying pulmonary cellular responses to AgNPs.

It is widely held that nanomaterials induce cytokine signal transduction, leading to a range of *in vitro* studies focused on the advantages of AgNPs for providing an anti-inflammatory effect and their role in wound healing by lowering the concentrations of inflammatory mediators such as interleukin 1 and interferons [8, 9, and 10]. Several researchers have investigated the effects of AgNPs on the bacterial wall and how AgNPs inhibit bacterial growth [11, 12, and 13]. However, despite these studies, the exact mechanism underlying the anti-inflammatory effect of AgNPs is not clearly understood. By the same way, we thought that AgNPs might have an anti-apoptotic effect along with its anti-inflammatory effect and we established this study to focus on the effect of AgNPs on the apoptosis as one of the most important cellular responses.

Tumor necrosis factor-α (TNFα) is a major pro-inflammatory cytokine and is usually detected in the early stage of cell inflammation. TNFα has many known signal transduction pathways such as NF-kB activation [14], MAPK activation [15], and the induction of cell death [16]. Moreover, TNFα induces apoptosis through the production of reactive oxygen species [17] and by activating caspase [18]. So, in this study, we used TNFα to induce apoptosis in lung epithelial cells and study the effect of AgNPs on the TNFα-induced apoptosis.

Briefly, our study investigated the effect of AgNPs on the cellular response to TNFα and the molecular mechanism of this response. The results indicate that AgNPs reduce the cell surface TNFR1 localization, suggesting that the reduction of surface TNFR1 reduces the cellular response to TNFα, resulting in an anti-apoptotic effect.

## 2. Material and methods

### 2.1. Cell culture

Human pulmonary epithelial cell line NCI-H292 (ATCC CRL-1848TM) cells were cultured at 37 °C in a 5% CO_2_ incubator in RPMI-1640 medium (L-glutamine with phenol red, Nacalai Tesque, Japan) supplemented with 10% (v/v) heated fetal bovine serum (HFBS, Biowest, USA), 100-μg/mL penicillin, and 10-μg/mL streptomycin (Nacalai Tesque, Japan). Cells were subcultured every three days.

### 2.2. Silver nanoparticles (AgNPs)

Polyvinylpyrrolidone (PVP)-coated AgNPs (Sigma-Aldrich, Cat. No. 576,832) have a particle size less than 100 nm. AgNPs were slightly aggregated, almost spherical, and the mean size was approximately 50–90 nm as measured using a transmission electron microscope (TEM; JEM-2000FX, JEOL, Japan). The average hydrodynamic diameter of AgNPs in  de-ionized water was 170 ± 50 nm and the polydispersity index was 0.202. The zeta potential of AgNPs in water was −53.2 mV as measured by electronic light scattering (zeta potential and particle size analyzer ELSZ-2000, Otsuka Electronics, Japan).

### 2.3. Tumor necrosis factor-α (TNFα)

Recombinant human TNFα (Peprotech, USA) was reconstituted in water to 0.1 mg/mL. The required further dilutions were prepared using endotoxin-free culture medium (RPMI-1640) containing a carrier protein.

### 2.4. Cell viability assay

The viability of NCI-H292 cells was measured using a CellTiter-Glo® luminescent cell viability assay (Promega, USA) according to the manufacturer’s protocol. Cells were seeded at a density of 1 × 104 cells/well in an opaque 96-well plate and incubated at 37 °C and 5% CO_2_ for 24 h, then the cells were exposed to different concentrations of AgNPs (0, 5, 10, 25, 50, 75, and 100 µg/mL) or different concentrations of TNFα (0, 10, 20, and 40 ng/mL) for 24 h. CellTiter-Glo® reagent was added to each well and the number of viable cells was measured based on quantification of adenosine triphosphate (ATP) using a luminometer (TECAN, Japan).

### 2.5. Cellular uptake assay

NCI-H292 cells were seeded at a concentration of 8 × 104 cells/well in a 24-well plate (Costar, USA). After 24 h of incubation, the cells were exposed to different concentrations of AgNPs (2.5, 5, 10, and 20 µg/mL). Other plates were exposed to AgNPs (5 µg/mL) and/or TNFα (20 ng/mL). After 24 h of exposure, the cells were washed twice with phosphate buffered saline (PBS, Sigma) and collected by trypsinization using trypsin EDTA (Wako, Japan) and centrifugation. Then, the cells were resuspended in 1 ml PBS supplemented with 6% HFBS and kept on ice until analysis.

The percentage of cells taking up AgNPs was analyzed using a flow cytometer (FACS, SP6800 spectral analyzer, Sony Biotechnology, Japan). Forward scatter (FSC) is the laser light scattered at narrow angles to the axis of the laser beam and is proportional to the cell size. Side scatter (SSC) is the laser light scattered at a 90° angle to the axis of the laser and is proportional to the intracellular density, which is increased by the uptake of nanoparticles. The mean SSC for each group of cells was calculated depending on the peak intensities of treated cells compared to the control cells using the software supplied with the instrument.

### 2.6. Apoptosis assay

NCI-H292 cells were seeded, treated, and collected using the same method described for the cellular uptake assay, and then an Annexin V-FITC apoptosis detection kit (Sigma Aldrich, USA) was used according to the manufacturer’s protocol to determine the degree of apoptosis. Cells were suspended in 500-µL 1× PBS supplemented with 6% HFBS, 5-µL Annexin V-FITC conjugate, and 10-µL propidium iodide solutions, then incubated for 10 min at room temperature and protected from light.

The fluorescence intensities of the cells were determined immediately using the SP6800 flow cytometer. Cells early in the apoptotic process are stained only with the Annexin V-FITC conjugate, healthy cells are not stained by either Annexin V-FITC conjugate or by propidium iodide, whereas late apoptotic cells are stained by both dyes. The percentage of cells in each category was determined using quadrant diagrams of treated-cell gates and compared to the control-cell gates using the software supplied with the flow cytometer.

### 2.7. Gene expression analysis by real-time (RT) PCR

For mRNA expression analysis of interleukin 17c (IL-17c), NCI-H292 cells were seeded at a concentration of 4 × 105 cells/60 mm cell culture dish. After 24 h of incubation, the cells were exposed to AgNPs (5 µg/mL) and/or TNFα (20 ng/mL). After 24 h of exposure, the cells were detached by trypsinization and collected by centrifugation, and then the total cellular RNA was extracted using an RNeasy kit (Qiagen, USA) according to the manufacturer’s protocol. An aliquot (1 µg) of the extracted total RNA was reverse transcribed into cDNA using a RT2 First Strand kit (SABiosciences/Qiagen, USA). The PCR primer for human IL-17c was purchased from SABiosciences/Qiagen. The reaction mixture was composed of 12.5-µL RT2 SYBR Green qPCR Master Mix (SABiosciences/Qiagen), 1-µL 10-µM gene-specific RT2 qPCR forward and reverse primers, 2-µL cDNA, and nuclease free water to a final volume of 25 µL. Glyceraldehyde-3-phosphate dehydrogenase (GAPDH) was used as a house-keeping gene to normalize the data. RT-PCR analysis was performed using an ABI PRISM 7000 sequence detection system (Applied Biosystems, Singapore) and the thermocycling conditions were 95 °C for 10 min, followed by 40 cycles of 95 °C for 15 s and 60 °C for 1 min.

### 2.8. Immunostaining and confocal laser scanning microscopy

To localize tumor necrosis factor receptor 1 (TNFR1), NCI-H292 cells were plated in a CELLview cell culture dish (Greiner Bio-one North America Inc., USA) at a density of 1.5 × 104 cells/compartment and incubated for 24 h. The cells were exposed to AgNPs (5 µg/mL) and/or TNFα (20 ng/mL) for 24 h. Then, the cells were washed with 1× PBS (Sigma), fixed with 4% formaldehyde solution in PBS (Wako, Japan) for 10 min at room temperature, permeabilized with 0.1% Triton X-100 for 5 min, and then blocked with 10% normal goat serum in PBS for 1 h. The cells were then incubated overnight at 4 °C with rabbit polyclonal anti-TNF receptor 1 antibody (Abcam, UK) followed by incubation with labeled goat anti-rabbit IgG H&L (Alexa Fluor 488) (Abcam, UK) for 1 h at room temperature. Nuclear DNA was stained with DAPI (4′, 6-diamidino-2-phenylindole) (Dojindo, Japan) for 5 min at room temperature. Microscopic images were acquired using a confocal laser-scanning microscope (LSM510 META, Carl Zeiss Inc., Germany) with a 63 × 1.4 Plan-Apochromat oil immersion lens.

### 2.9. Statistical analysis

Data were subjected to statistical analysis using Student’s t-test. Differences between means of different groups were determined using one-way ANOVA with Duncan multiple comparison tests. The values of *P* < 0.05 were considered statistically different. Data are presented as mean ± standard deviation (SD) with at least three independent replicates (*n* ≥ 3).

## 3. Results

### 3.1. Effect of AgNPs and TNFα on cell viability

**Figure 1.**
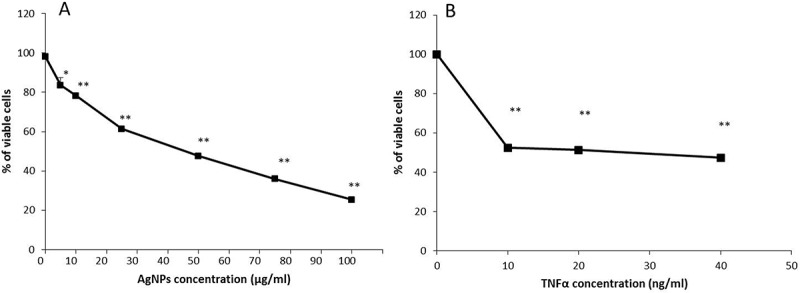
**Effect of AgNPs and TNFα on the viability of NCI-H292 cells. (A)** The viability of cells exposed to AgNPs at concentrations of 0, 5, 10, 25, 50, 75, and 100 µg/mL. **(B)** The viability of cells exposed to TNFα at concentrations of 0, 10, 20, and 40 ng/mL. Cells were exposed to AgNPs and TNFα for 24 h, then cell viability was determined using a CellTiter-Glo® luminescent cell viability assay. The results are shown as mean ± SD, *n* ≥ 3, for each group; *0.01 < *P* < 0.05 and ** *P* < 0.01. * Represents significant difference compared to the control group.

AgNPs are known to have a cytotoxic effect on different cell lines, but our aim is to study the effect of AgNPs on the cellular response of TNFα, so we don’t need to induce a cytotoxic effect by the AgNPs itself, because of that we first needed to assess the cytotoxic effect of AgNPs and determine accurately a suitable concentration (resulting in a very low or no cytotoxic effect) to use in subsequent experiments. We therefore conducted a cell viability assay to determine the effect of different concentrations of AgNPs on the viability of NCI-H292 cells. As shown in Figure 1(a), the percentage of viable cells decreased as the concentration of AgNPs increased in a dose-dependent manner. The percentage of viable cells after 24 h exposure to 10, 25, 50, 75 or 100 µg/mL AgNPs was 78.2, 61.4, 47.5, 35.9, and 25.3%, respectively, showing that a higher concentration of AgNPs induced a higher cytotoxic effect. In addition, the lowest concentration of AgNPs (5 µg/mL) resulted in the highest percentage of viable cells (83.5%). Based on these data, a concentration of 5 µg/mL which induced the lowest cytotoxic effect was chosen to carry out the following experiments in this study.

TNFα is a major proinflammatory mediator and efficiently induces a cytotoxic effect in most cell lines [19]. In this study, we used TNFα to induce the apoptosis, therefore it was important to assess the cytotoxic effect of low concentrations of TNFα (10, 20, and 40 ng/mL) on the NCI-H292 cell line to choose a suitable concentration for subsequent experiments. As shown in Figure 1(b), the percentage of viable cells after 24 h exposure to TNFα significantly decreased to about 50%, even following exposure to the lowest concentration (10 ng/mL). The decrease was similar for all concentrations tested. Therefore, a concentration of 20 ng/mL – which induces the required cytotoxic effect – was selected as an average concentration for the following experiments conducted to determine the interference of AgNPs with TNFα in the cells.

### 3.2. Effect of TNFα on the cellular uptake of AgNPs

**Figure 2.**
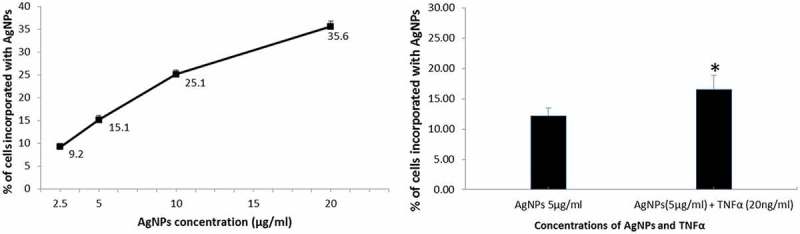
**Effect of TNFα on the cellular uptake of AgNPs. (A)** Percentage of cells incorporating AgNPs after exposure to 2.5, 5, 10, and 20 µg/mL of AgNPs. **(B)** Percentage of cells incorporating AgNPs after exposure to AgNPs (5 µg/mL) with and without TNFα (20 ng/mL). Cells were exposed to AgNPs and TNFα for 24 h, then cellular uptake was determined based on side scatter (SSC) using FACS. The results are shown as mean ± SD, *n* ≥ 3, for each group; *0.01 < *P* < 0.05.

The cellular uptake of nanoparticles is crucial in the cellular response to these nanoparticles, including apoptosis, inflammation, and many other responses. We therefore estimated the cellular uptake of AgNPs. First, we assessed the cellular uptake of different concentrations of AgNPs alone and the results are shown in Figure 2(a). AgNPs were taken up by the cells in a dose-dependent manner, and the percentage of cells incorporating AgNPs increased from 9.2% to 35.6% when the concentration of AgNPs increased from 2.5 to 20 µg/mL. Next, we assessed the effect of TNFα (20 ng/mL) on the cellular uptake of AgNPs (5 µg/mL) and observed that the cellular uptake of AgNPs increased significantly, from 12% to 16.5%, in the presence of TNFα, as shown in Figure 2(b). These results demonstrate that a higher concentration of AgNPs induces higher cellular uptake, and that TNFα plays a role in increasing the cellular uptake of AgNPs.

### 3.3. Interference of AgNPs with TNFα-induced apoptosis

**Figure 3.**
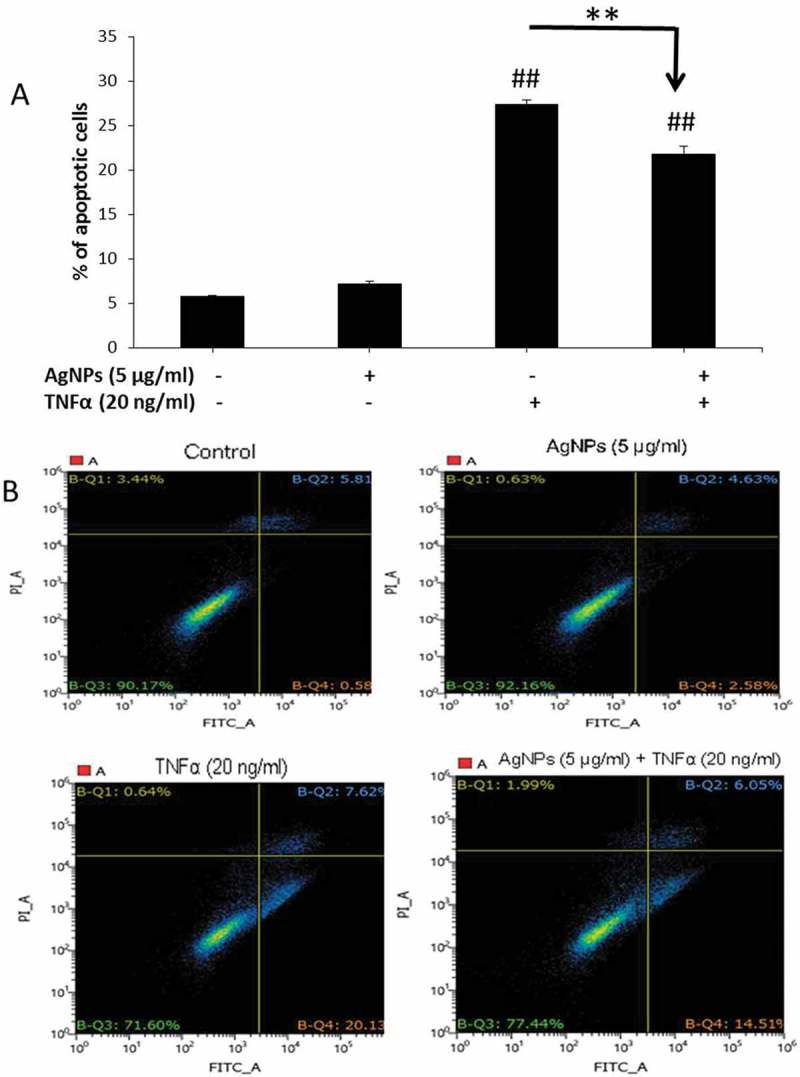
**Effect of AgNPs and TNFα on apoptosis in NCI-H292 cells. (A)** Percentage of apoptotic cells following 24 h exposure to AgNPs (5 µg/mL) only, TNFα (20 ng/mL) only, or both AgNPs (5 µg/mL) and TNFα (20 ng/mL) together as compared to control cells. Data were obtained by FACS measurements. **(B)** Representative quadrant diagrams for NCI-H292 cells exposed to AgNPs (5 µg/mL) and/or TNFα (20 ng/mL) for 24 h and stained using an Annexin V-FITC apoptosis detection kit. The samples were analyzed using FACS. The results are shown as mean ± SD, *n* ≥ 3, for each group; ** and ## means *P* < 0.01. ## Represents significant difference compared to the control group. ** represents significant difference compared to the marked corresponding group. (+) means with, (−) means without.

In the cellular inflammatory response, there are plenty of TNFα molecules as a major proinflammatory cytokine. Apoptosis is one of the most common cellular responses induced by TNFα and thus we conducted an apoptosis assay to understand how AgNPs affect this response. As shown in Figure 3(a), cells exposed to AgNPs (5 µg/mL) only did not show any apoptotic cells compared to the control group suggesting that this concentration of AgNPs does not exert any apoptotic response by its own action. Also, the percentage of apoptotic cells induced by TNFα (20 ng/mL) was the highest (27.39%) and significantly decreased, from 27.39% to 21.78%, upon exposure to AgNPs (5 µg/mL) for 24 h showing the anti-apoptotic effect of AgNPs. Figure 3(b) shows the quadrant diagrams obtained after staining cells with Annexin V-FITC conjugate (Annexin V) and propidium iodide (PI) and conducting flow cytometry. Healthy cells are shown in the lower-left quarter (negative for both stains), the lower-right quarter shows early apoptotic cells (positive for Annexin V and negative for PI), while the upper-right quarter shows late apoptotic cells (positive for both stains) and the upper-left quarter shows necrotic cells (negative for Annexin V and positive for PI). These results indicate that the significant decrease in the percentage of apoptotic cells induced by TNFα after exposure to AgNPs was mainly due to the significant decrease in early apoptotic cells (left-lower quarters of Figure 3(b)). These data demonstrated that the concentration of 5 µg/mL of AgNPs had no apoptotic effect but acted as an antagonist against TNFα-induced apoptosis.

### 3.4. Effect of AgNPs and TNFα on Il-17c gene expression

**Figure 4.**
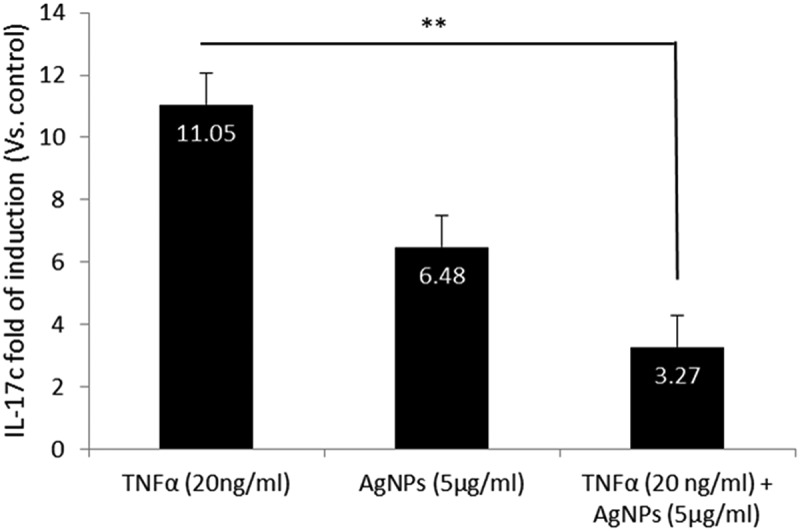
**Effect of AgNPs and TNFα on IL-17c gene expression**. NCI-H292 cells were exposed to AgNPs (5 µg/mL) and/or TNFα (20 ng/mL) for 24 h. IL-17c mRNA expression was measured using a real-time (RT) PCR technique. The results are shown as mean ± SD, *n* ≥ 3, for each group; ** *P* < 0.01 and represents significant difference compared to the marked corresponding group.

Many studies suggest the role of IL-17 family members in apoptosis and inflammation; for example, IL-17c regulates the action of IL-1β and TNFα by cooperation with other IL-17 family members [20]. We therefore analyzed the gene expression of IL-17c as a proinflammatory cytokine after exposing cells for 24 h to AgNPs (5 µg/mL) only, TNFα (20 ng/mL) only, and both together. The results showed that the expression of IL-17c increased 11-fold after exposure to TNFα compared to the control group. Moreover, this high fold induction was significantly decreased after exposure to both AgNPs and TNFα, as shown in Figure 4. These results demonstrate the antagonistic effect of AgNPs against the high expression of IL-17c induced by TNFα.

### 3.5. Localization of tumor necrosis factor-α receptor 1 (TNFR1)

**Figure 5.**
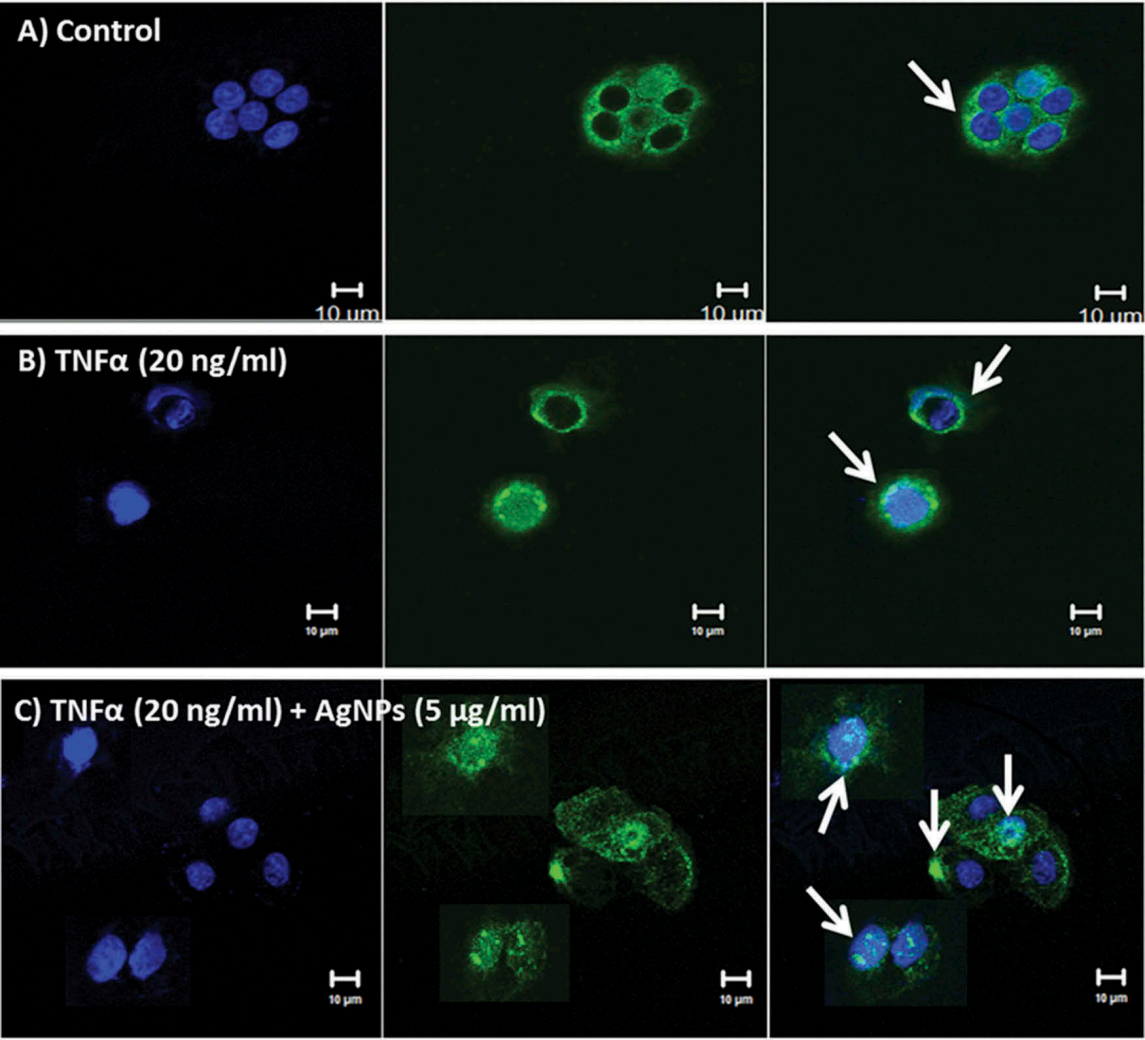
**Localization of TNFR1 in NCI-H292 cells using a confocal microscope**. Blue shows the nucleus, green shows the receptor (TNFR1), and blue and green together are the merged form. White arrows show TNFR1. **(A)** The control NCI-H292 cells, without exposure to AgNPs or TNFα, showing that TNFR1 is homogenously distributed on the cell membrane. **(B)** The NCI-H292 cells exposed to TNFα (20 ng/mL) for 24 h, showing that TNFR1 is slightly aggregated and scattered over the entire cell membrane. **(C)** The NCI-H292 cells exposed to both TNFα (20 ng/mL) and AgNPs (5 µg/mL) for 24 h, showing that TNFR1 localizes inside the cells, with very few receptors scattered on the cell membrane. Scale bar is 10 µm for all panels.

TNFR1 is a major receptor for TNFα, mediates apoptosis, and functions as a regulator of inflammation [21]. Our cellular uptake assay results indicated that TNFα increases the cellular uptake of AgNPs. We demonstrated the localization of TNFR1 by immunofluorescence staining using confocal microscopy. The results revealed that TNFR1 is homogenously distributed on the cell membrane of control cells, as shown in Figure 5(a). In contrast, TNFR1 was slightly aggregated and scattered over the entire cell membrane of cells exposed to TNFα (20 ng/mL), as shown in Figure 5(b), whereas in cells exposed to both TNFα (20 ng/mL) and AgNPs (5 µg/mL), TNFR1 was localized inside the cells and very few receptors were scattered on the cell membrane, as shown in Figure 5(c). The results indicated that AgNPs reduced the expression level of cell surface TNFR1 and suggested that this reduction in surface TNFR1 reduced the cellular response to TNFα, resulting in an anti-apoptotic effect.

## 4. Discussion

AgNPs have potential antimicrobial and anti-inflammatory effects, as reported in many previous studies, but very few studies have reported details about the molecular mechanism of this anti-inflammatory and protective effect of AgNPs. In this study, we demonstrated that AgNPs have an anti-apoptotic effect by disturbing the pathway of TNFR1 in lung epithelial cells.

A previous study of the protective effect of AgNPs reported the overexpression of HSP70 in Clone 9 cells [22]. Our aim was to obtain more details about the molecular mechanism underlying the protective effect of AgNPs and thus we used a low concentration of AgNPs (5 µg/mL) so as to not induce a cytotoxic effect. In addition, we used TNFα (20 ng/mL) as an apoptotic agent to investigate the interference between AgNPs and TNFα. Interestingly, our results showed that TNFα induced more cellular uptake of AgNPs (Figure 2(b)) and that a low concentration of AgNPs enhanced the protective effect against apoptosis induced by TNFα (Figure 3). IL-17c is a proinflammatory cytokine and a regulator for TNFα action in apoptosis, and thus the results of IL-17c gene expression analysis (Figure 4) were consistent with the results of the apoptosis assay, confirming the anti-apoptotic effect of a low concentration of AgNPs against TNFα-induced apoptosis.

**Figure 6.**
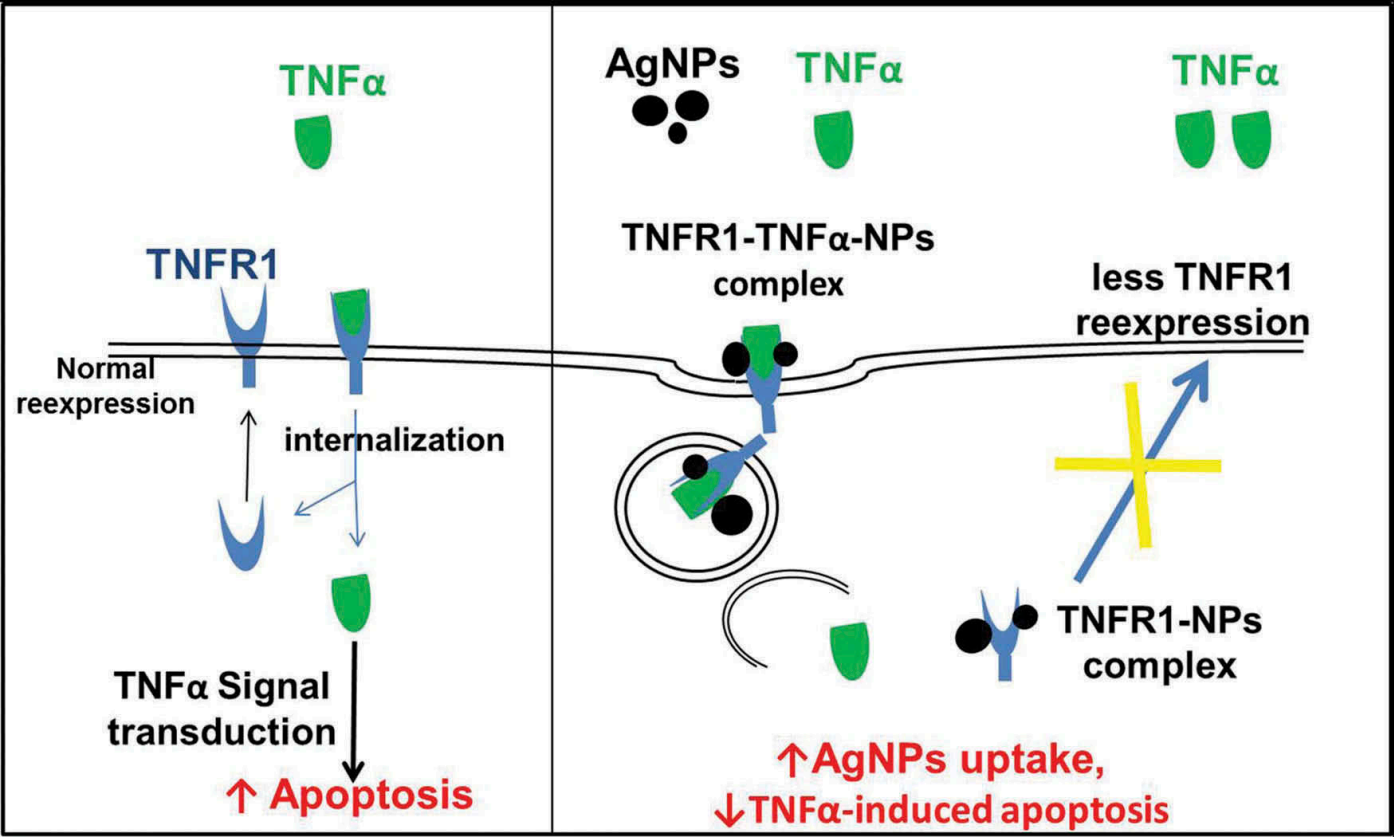
Molecular mechanism explaining why the cellular uptake of AgNPs increases in the presence of TNFα, and how the AgNPs reduce the apoptosis induced by TNFα.

The localization of TNFR1 in lung epithelial cells after their exposure to both AgNPs and TNFα showed its presence mainly inside the cells, with very few receptors scattered on the cell membrane. In contrast, TNFR1 was homogenously distributed on the cell membrane of the control cells and cells exposed to TNFα only. These results guided us to consider the molecular mechanism shown in Figure 6. In cells exposed to TNFα only, TNFα binds to TNFR1 specifically and enters the cells, and then TNFα is released from its receptors, freeing the receptors to return to the cell membrane to bind more TNFα molecules. This cycle would induce TNFα-signal transduction, leading to apoptosis. In contrast, in cells exposed to both TNFα and AgNPs, the nanoparticles non-specifically bind to TNFR1 and TNFα binds specifically with the same receptor, forming a TNFR1-TNFα-AgNPs complex which then enters the cells by receptor-mediated endocytosis. TNFα is then released from the receptor and induces apoptosis. The receptors might still bind to AgNPs, thus disturbing the receptor’s shape, molecular weight, and characteristics, leading to disturbance of its normal pathway of being recycled to the cell membrane, resulting in less TNFR1 on the cell membrane and more inside the cells, as shown by confocal microscopy results (Figure 5(c)). This molecular mechanism explains how TNFR1 would play a role in increasing the cellular uptake of AgNPs and reducing TNFα-induced apoptosis as a result. The mechanism clarify that the NPs — TNFR1 complex hinder the re-expression pathway of the receptors on the cell membrane leading to decrease in the TNFα signal transduction and its apoptotic effect.

AgNPs have a potent protective anti-inflammatory effect; however, many *in vivo* studies suggested the toxic effect of AgNPs. So AgNPs is considered a double-edged sword depending on many factors such as size, concentration, route of administration and the purpose of use. From the *in vitro* studies, we could pick one factor and investigate its effect; in this study, we focused on the concentration recommending that the lower concentration of AgNPs has a lower cytotoxic effect and an anti-apoptotic effect too. We could not decide whether AgNPs were toxic or non-toxic without specifying various factors.

## 5. Conclusion

In this study, we confirmed the anti-apoptotic effect of the AgNPs in NCI-H292 cells and suggested a mechanism for this effect. This mechanism involves contact between the cells and nanoparticles by reducing the expression of TNFR1 and thus decreasing TNFα-signal transduction, leading to an anti-apoptotic effect with increasing cellular uptake of AgNPs.
