# Supplementary material for: Silver nanoparticles reduce the apoptosis induced by tumor necrosis factor-α
Source: Sci Technol Adv Mater. 2018 Jul 16;19(1):526–34. doi: 10.1080/14686996.2018.1487761 (PMC6052409; doi:10.1080/14686996.2018.1487761)
Supplement: supplemntal_fig..doc [file TSTA_A_1487761_SM6424.doc]

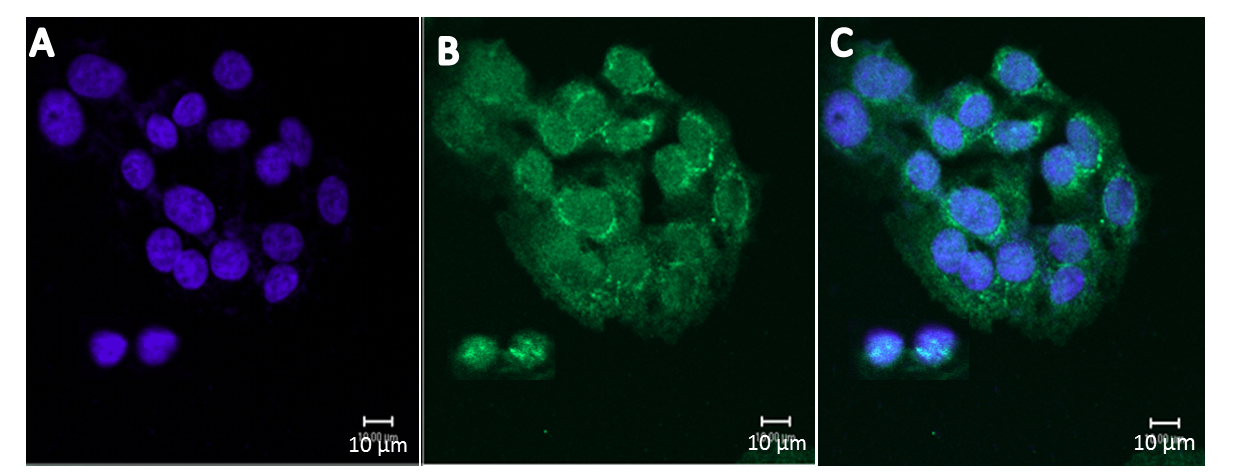


Supplemental figure. Localization of TNFR1 in NCI-H292 cells using a confocal microscope. The NCI-H292 cells exposed to AgNPs (5 µg/ml) for 24 h, showing that TNFR1 localizes inside the cells, with very few receptors scattered on the cell membrane. (A) Blue shows the nucleus, (B) green shows the receptor (TNFR1), and (C) blue and green together are the merged form. White arrows show TNFR1. Scale bar is 10 µm for all views.

Supplemental figure. Localization of TNFR1 in NCI-H292 cells using a confocal microscope. The NCI-H292 cells exposed to AgNPs (5 µg/ml) for 24 h, showing that TNFR1 localized inside the cells, with very few receptors scattered on the cell membrane. **(A)** Blue shows the nucleus, **(B)** green shows the receptor (TNFR1), and **(C)** blue and green together are the merged form. White arrows show TNFR1. Scale bar is 10 µm for all views.
